# Effectiveness of Calorie Restriction for Weight Loss in Type 2 Diabetes Mellitus: A Systematic Review

**DOI:** 10.7759/cureus.78348

**Published:** 2025-02-01

**Authors:** Tarig A Mohamed, Molly Mckeown, Manish Saxena

**Affiliations:** 1 Family Medicine, Primary Health Care Corporation, Doha, QAT; 2 Nursing, Crocus Medical Practice, Saffron Walden, GBR; 3 Research, Barts Health National Health Service (NHS) Trust, London, GBR

**Keywords:** body mass index (bmi), calorie restriction, hemoglobin a1c (hba1c), low calorie diet, remission, type 2 diabetes, weight loss/reduction

## Abstract

The effectiveness of calorie restriction (CR) for weight loss in type 2 diabetes mellitus (T2DM) has not been thoroughly studied. This review aims to evaluate CR’s short- and long-term effectiveness for weight loss and its impact on cardiometabolic parameters in T2DM. Following Preferred Reporting Items for Systematic Reviews and Meta-Analyses (PRISMA) guidelines, Medline Complete, Embase, Cumulative Index to Nursing and Allied Health Literature (CINAHL), and Cochrane Library databases were searched to identify studies up to June 7, 2024. Furthermore, a reference search was conducted. Randomized controlled trials involving adults with T2DM examining CR and reporting weight changes were included. The revised Cochrane risk-of-bias tool was used to assess the study’s quality. A narrative synthesis was used to analyze the findings. Eleven studies, with 1,554 participants, were included; all had a low risk of bias. The intervention group participants’ mean baseline weight and Body Mass Index were 93.3 kg and 32.7 kg/m², respectively. Interventions used included total diet replacement (TDR) and very low- and low-calorie diets lasting 12 weeks to two years. CR with TDR resulted in >12% weight loss. Additionally, CR improved cardiometabolic parameters; glycated hemoglobin (HbA1c) decreased to ≤6.5%; diabetes remission was achieved in 19% to 83%; high-density lipoprotein significantly increased; and triglyceride, systolic, and diastolic blood pressure significantly decreased. In conclusion, CR effectively reduces weight and improves cardiometabolic markers in T2DM. However, large long-term studies addressing CR in T2DM are lacking, which challenges drawing firm conclusions. This highlights the need for further research to address this gap.

This review is registered in the International Prospective Register of Systematic Reviews (PROSPERO) under the registration number CRD42024573505.

## Introduction and background

Type 2 diabetes mellitus (T2DM) is a significant global health challenge with a rising prevalence. In 2021, an estimated 537 million people worldwide were living with diabetes mellitus, and projections indicate that this number will increase to 643 million by 2030 and 783 million by 2045 [1]. Over 90% of these cases are attributed to T2DM [1]. Obesity and overweight are significant risk factors for the development of T2DM [2]. Notably, since 1990, rates of overweight and obesity have surged, doubling among adults and quadrupling among adolescents [3]. As of 2022, 43% of adults were classified as overweight, and 16% were living with obesity worldwide, further exacerbating the global diabetes burden [3].

The risk of developing T2DM can be mitigated with lifestyle interventions, including weight loss [4]. Moreover, weight loss can improve glycemic control and facilitate diabetes remission [5-7]. Therefore, experts recommend shifting the focus of T2DM management from glycemic control to obesity management [8]. This is in accordance with the recommendations of the American Diabetes Association (ADA) [6] and the European Association for the Study of Diabetes (EASD) [5].

Calorie restriction (CR) is defined as reducing daily calorie intake below the typical average without depriving the body of essential nutrients [9]. It is important to distinguish CR from intermittent fasting, which involves alternating periods of eating and fasting without specifically restricting calorie intake. This review focuses exclusively on CR interventions.

CR can be implemented in various ways and applied either intermittently or continuously (daily CR). The most intensive form involves replacing regular meals with formula-based or calorie-restricted meals. However, CR can also be achieved by monitoring and reducing the calorie content of normal meals. This review includes studies that represent different forms of CR. A distinction is made between a very low-calorie diet (VLCD) and a low-calorie diet (LCD) based on the degree of CR, with VLCD defined as having a daily calorie intake of ≤800 kcal [10].

Although some systematic reviews have explored related topics, such as the effectiveness of CR [7,11] or low-carbohydrate diets [12] in achieving T2DM remission, there is a lack of reviews focusing specifically on the effectiveness of CR for weight loss in T2DM. The need for this review is evident, as evidence supporting CR’s role in weight loss has accumulated since 1998 when a study reported weight loss with a VLCD [13]. Recent studies have further contributed to this topic, highlighting its relevance [14-20]. Therefore, this review aims to address this gap by evaluating the effectiveness of CR, compared to standard care, for weight loss and other related health outcomes in T2DM patients.

There are two objectives for this review: (1) to assess the effectiveness of CR in promoting weight loss in T2DM patients, considering both short- and long-term effectiveness, and (2) to assess the impact of CR on cardiometabolic markers, including blood pressure (BP), glycated hemoglobin (HbA1c) levels, and lipid parameters.

This review is registered in the International Prospective Register of Systematic Reviews (PROSPERO) under the registration number CRD42024573505.

## Review

Methods

Search Strategy

This systematic review followed the Preferred Reporting Items for Systematic Reviews and Meta-Analyses (PRISMA) guidelines [21]. The primary author (TM) conducted a comprehensive search across Medline Complete, Embase, Cumulative Index to Nursing and Allied Health Literature (CINAHL), and Cochrane Library, applying consistent key terms across all databases. No restrictions were placed on language, region, or publication date, and the search included all publications from the databases’ inception to June 7, 2024. To ensure comprehensive coverage, the primary author conducted a reference search using an artificial intelligence tool, CitationChaser [22].

The Boolean search phrase used was: (“Caloric Restriction” OR “Calorie Restricted Diet” OR “Calorie Restricted Diets” OR “Calorie Restricted Diet” OR “Caloric Restricted” OR “Low-Calorie Diet” OR “Low-Calorie Diets”) AND (“Noninsulin-Dependent Diabetes Mellitus” OR “Non Insulin Dependent Diabetes Mellitus” OR “Non-Insulin-Dependent Diabetes Mellitus” OR “Type II Diabetes Mellitus” OR “NIDDM” OR “Type 2 Diabetes Mellitus” OR “Noninsulin-Dependent Diabetes Mellitus” OR “Noninsulin Dependent Diabetes Mellitus” OR “Type 2 Diabetes” OR “Adult-Onset Diabetes Mellitus” OR “Adult Onset Diabetes Mellitus”).

Study Selection

Two authors (TM, MM) independently screened the identified studies by reviewing titles and abstracts against predefined inclusion and exclusion criteria. The focus was on identifying randomized clinical trials (RCTs) involving adult participants with T2DM who received a form of CR as the sole intervention. Eligible studies had to report outcomes related to weight or Body Mass Index (BMI) changes and be published in English to ensure high-quality, peer-reviewed sources. Any disagreements during the selection process were resolved through discussion between the screening authors. Rayyan, a web-based tool designed to screen and select studies for systematic reviews was used to facilitate the selection process [23]. However, their auto-selection feature was not utilized, and the two authors completed the process manually. The list of inclusion and exclusion criteria used in the selection process can be found in Table 1.

**Table 1:** Inclusion and exclusion criteria used in the selection process. RCT, randomized controlled trial; T2DM, type 2 diabetes mellitus; BMI, Body Mass Index; CR, calorie restriction.

Inclusion criteria	Exclusion criteria
The study must be an RCT.	Studies that were non-RCTs.
The study population must consist of adults (≥18 years).	Studies where the population is not adults.
The study population must be patients with T2DM.	Studies involving populations with diabetes mellitus other than T2DM.
The intervention must be a calorie-restricted diet.	Studies where the intervention is not a calorie-restricted diet.
The outcome must include the change in weight or BMI.	Studies where the study outcomes do not report weight change.
The study must be published in the English language.	Studies not available in the English language.
	Studies where CR was combined with other interventions.

Data Extraction

Relevant data were extracted by the primary author (TM) from the identified RCTs using a pre-developed template. Key information on the primary and secondary outcomes was systematically collected from the main text and supplementary materials, with missing information noted in the text or tables. The template used for data extraction is presented in the Appendix. The mean age, BMI, weight, male percentage, and their respective standard errors of the mean (SEM) were calculated for the intervention groups across the studies. When not reported, weight and HbA1c mean differences and percentage changes were calculated for the intervention groups. The percentage changes are illustrated in the graphs provided in the Results section.

Quality Assessment

The selected studies were assessed for risk of bias by the primary author (TM) using the revised Cochrane risk-of-bias tool for randomized trials (RoB-2) [24]. This tool evaluates the risk of bias across five domains: bias arising from the randomization process, deviations from intended interventions, missing outcome data, measurement of the outcome, and selection of the reported result.

Data Analysis

A narrative synthesis was employed to analyze the data. Mean changes in weight, BMI, HbA1c, lipid parameters, and BP following CR were assessed to evaluate both the short- and long-term effectiveness of CR. The precision measures of the mean varied among studies and were presented without modifications, with definitions of which measures were employed. The studies were grouped based on the intervention used, study duration, follow-up period, study characteristics, and outcomes, facilitating the comparison of findings. No statistical tests were performed in the analysis; heterogeneities were investigated by examining variations in study characteristics, including sample size, intervention type, duration, and outcome measures.

Results

Study Selection and Screening Process

**Figure 1:**
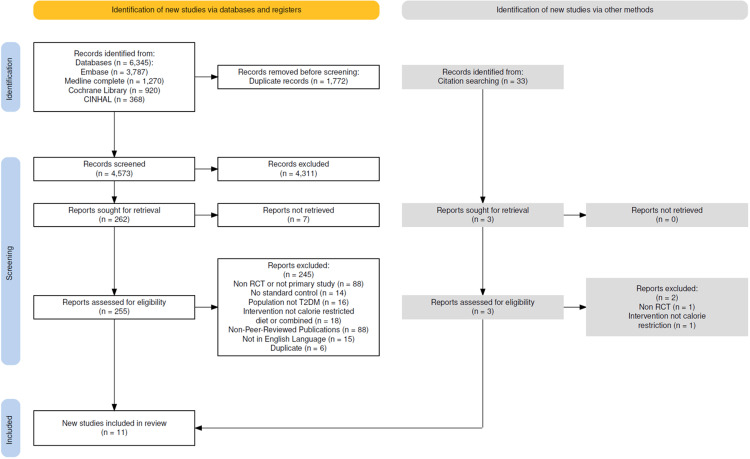
PRISMA flow diagram of study selection process, RCT: randomized control trial. PRISMA, Preferred Reporting Items for Systematic Reviews and Meta-Analyses.

The database searches identified 6,345 records, including 1,772 duplicates. Two authors (TM, MM) independently screened the remaining 4,573 records. During this phase, 4,311 records were excluded based on the title and abstract, leaving 262 reports for full-text retrieval. Seven studies could not be retrieved, so 255 were assessed for eligibility, of which 245 were excluded, leaving 10 studies eligible for inclusion in the review. The selection process and reasons for exclusion are presented in the PRISMA flow diagram, Figure 1.

Thirty-three additional studies were identified through the reference search. Three were retrieved for full-text screening, and one was selected, bringing the total number of studies in the review to eleven.

Fourteen studies were excluded despite meeting the inclusion criteria because they did not compare CR to standard care; instead, they compared different forms of CR or CR to surgical interventions, which did not directly address the review question. Therefore, their inclusion would not contribute to answering the review question.

Study Characteristics

With one exception [13], the selected studies were published recently, between 2017 and 2024. They were conducted in different countries: three in the United Kingdom [16,17,25], three in China [19,20,26], two in Italy [14,15], and one in South Africa [27], Thailand [18], and the United States [13]. The characteristics of the selected studies are summarized in Table 2.

**Table 2:** Characteristics of selected studies. INT, intervention group; BMI, Body Mass Index; RCT, randomized controlled trial; PROBE, prospective, randomized, open-label, blinded endpoint; FU, follow-up; SD, standard deviation; SEM, standard error of the mean; IQR, interquartile range; 95% CI, 95% confidence interval. * Three months intervention period extended, if the participant wishes, to five months; † Mean (SEM); ‡ For all participants; § Median (IQR); || Median (95% CI).

S. No.	Study	Study type	Number of participants		Age in the INT	INT males percentage	Baseline BMI INT	Duration of the INT	Follow-up duration	Comparator group
			Total	INT	Mean, years (SD)		Mean, kg/m² (SD)			
1	Lean et al. (2018) [25], United Kingdom	Open-label, cluster-randomized trial	306	137	52.90 (7.60)	55.70%	35.00 (4.50)	3–5 months^*^	12 months	Best-practice care by standard guidelines
2	Yang et al. (2023) [19], China	RCT	72	32	52.20 (7.38)	63.40%	24.23 (2.58)	3 months	12 months	ad libitum diet throughout​
3	Williams et al. (1998) [13], United States; (1-day group)	RCT	54	16	51.40 (7.90)	50.00%	35.40 (5.40)	20 weeks	No FU	Standard Behavioral Therapy (SBT) Group
	Williams et al. (1998) [13], United States; (5-day group)			15	50.30 (8.60)	39.00%	37.30 (4.80)			
4	Umphonsathien et al. (2022) [18], Thailand; (2-day group)	RCT	40	14	49.50 (7.20)^†^	14.50%	29.90 (1.60)^†^	20 weeks	No FU	Normal diet of 1,500-2,000 kcal/day
	Umphonsathien et al. (2022) [18], Thailand; (4-day group)			14	47.60 (7.90)^†^	50.00%	31.00 (1.60)^†^			
5	Ruggenenti et al. (2022) [15], Italy	PROBE trial	103	53	64.90 (7.50)	78.60%	32.30 (3.70)	24 months	No FU	Standard diet according to guidelines
6	Ruggenenti et al. (2017) [14], Italy	RCT	74	34	59.80 (7.10)^‡^	75.70%^‡^	30.00 (3.90)	6 months	No FU	Standard diet according to guidelines
7	Mollentze et al. (2019) [27], South Africa	RCT	18	9	55.64 (7.72)	100.00%	41.30 (4.41)	6 months	No FU	Standard medical nutrition intervention
8	Brown et al. (2020) [16], United Kingdom	RCT	90	45	58.50 (50.10 – 64.20)^§^	43.00%	36.60 (5.10)	12 weeks	12 months	Standardized dietetic care involving individualized dietary advice to achieve a modest calorie deficit
9	Gulsin et al. (2020) [17], United Kingdom	PROBE trial with nested case-control study	87	24	50.50 (6.50)	59.00%	35.20 (33.50 - 40.30)^||^	12 weeks	No FU	Routine care as per National Institute for Health and Care Excellence (NICE) guidance
10	Hu et al. (2019) [26], China	RCT	384	128	53.10 (10.80)	50.00%	29.20 (NR)	6 months	No FU	Usual care with a standardized diet
11	Li et al. (2024) [20], China	RCT two-center, open-label, three-arm, parallel-group	326	109	52.99 (8.21)	63.30%	27.59 (2.48)	12 weeks	36 weeks	Routine lifestyle education based on healthy diet and exercise guidelines

Together, the included studies had a population of 1,554 participants, with 615 in the intervention groups. However, the sample sizes were variable, ranging from 18 to 384. The males represented 57.1% ± 5.8% (SEM) of the intervention groups. The mean age of the intervention groups’ participants was 54 years ± 1.3 years (SEM), the mean weight was 93.3 kg ± 4.8 kg (SEM), and the mean BMI was 32.7 kg/m² ± 1.3 kg/m² (SEM). There was no significant difference in the baseline characteristics between the intervention and control groups.

The intervention duration ranged from 12 weeks to two years; four studies had post-intervention follow-up [16,19,20,25]. For details on the description of the intervention, see Table 3.

**Table 3:** Description of the interventions in the selected studies. CMNT, Chinese medical nutrition therapy; VLCD, very low-calorie diet; MRP, meal replacement plan.

S. No.	Study	Description of intervention
1	Lean et al., (2018) [25], United Kingdom	Low-calorie formula diet during total diet replacement, with gradual food reintroduction. The low-calorie formula diet used was Counterweight Plus, a commercial total diet replacement formula designed to provide 825-853 kcal/day during the total diet replacement phase, followed by gradual food reintroduction.
2	Yang et al. (2023) [19], China	The CMNT group consumed meals consisting of fruit and vegetable gruel, solid beverages, composite nutritional rice, and meal replacement biscuits during fasting days​ ensuring controlled caloric intake during these periods. The kit contains approximately 840 kcal/day.
3	Williams et al. (1998) [13], United States; (1-day group)	Participants consumed 400–600 kcal/day during 5 consecutive days in week 2, then 1 day/week for 15 weeks, for a total of 20 VLCD days. Participants used high-quality protein sources, such as lean meat, fish, or fowl, and portion-controlled low-calorie diet entrees. The food was provided to participants to ensure compliance during the VLCD days.
	Williams et al. (1998) [13], United States; (5-day group)	Participants consumed 400–600 kcal/day for 5 consecutive days during weeks 2, 7, 12, and 17, for a total of 20 VLCD days. Participants used high-quality protein sources, such as lean meat, fish, or fowl, and portion-controlled low-calorie diet entrees. The food was provided to participants to ensure compliance during the VLCD days.
4	Umphonsathien et al. (2022) [18], Thailand; (2-day group)	Participants followed a VLCD diet (600 kcal/day) for 2 non-consecutive days per week, with unrestricted eating on the other days.
	Umphonsathien et al. (2022) [18], Thailand; (4-day group)	Participants followed a VLCD diet (600 kcal/day) for 4 non-consecutive days per week, with unrestricted eating on the other days.
5	Ruggenenti et al. (2022) [15], Italy	Participants followed a calorie-restricted diet with a 25% reduction in daily caloric intake.
6	Ruggenenti et al. (2017) [14], Italy	Participants followed a calorie-restricted diet with a 25% reduction in daily caloric intake.
7	Mollentze et al. (2019) [27], South Africa	Participants followed a commercially available low-energy diet tailored for weight loss in type 2 diabetes patients.
8	Brown et al. (2020) [16], United Kingdom	800-850 kcal/day for 12 weeks, followed by a structured food reintroduction and weight maintenance phase. Participants used the Counterweight Plus formula, which provided 800-850 kcal/day for 12 weeks.
9	Gulsin et al. (2020) [17], United Kingdom	Low-energy meal replacement diet (MRP) (~810 kcal/day).
10	Hu et al. (2019) [26], China	Calorie-restricted diet, aiming for a weight loss of 5–10% caloric deficit of 500 kcal per day, compared with the standardized diet.
11	Li et al. (2024) [20], China	Two days per week (mostly consecutive) of energy restriction (790 kcal/day) using a low-energy formula diet, regular diet on the remaining 5 days.

The outcome measures and the “inclusion and exclusion” criteria varied across the selected studies; details of the primary and secondary outcome measures are presented in Table 4, and the inclusion and exclusion criteria used for participant selection in the studies are presented in Table 5.

**Table 4:** Primary and secondary outcome measures of the selected studies. HbA1c, glycated hemoglobin; BMI, Body Mass Index, HDL, high-density lipoprotein; LDL, low-density lipoprotein.

S. No.	Study	Primary outcome(s) assessed	Secondary outcome(s) assessed
1	Lean et al. (2018) [25], United Kingdom	Weight loss of 15 kg or more remission of diabetes (HbA1c less than 6.5% off antidiabetic medications for at least two months).	Quality of life (measured by the EuroQol 5 Dimensions visual analog scale). Serum lipids, physical activity, sleep quality, and blood pressure.
2	Yang et al. (2023) [19], China	Diabetes remission, defined as maintaining an HbA1c level of less than 48 mmol/mol (<6.5%) for at least three months after discontinuing all antidiabetic medications.	HbA1c levels, fasting blood glucose levels, blood pressure, body weight, quality of life, and medication costs.
3	Williams et al. (1998) [13], United States; (1-day group)	Weight loss glycemic control (fasting plasma glucose {FPG} and HbA1c levels).	Insulin levels lipid profiles (cholesterol, triglycerides).
	Williams et al. (1998) [13], United States; (5-day group)		
4	Umphonsathien et al. (2022) [18], Thailand; (2-day group)	Changes in glycemic control (plasma glucose and HbA1C levels). Rate of diabetes remission defined as an FPG level <126 mg/dL and an HbA1C level <6.5% in the absence of pharmacological therapy for diabetes, at the end of the study.	Changes in insulin secretion, insulin sensitivity, anthropometric parameters, cardiovascular risk factors, and quality of life.
	Umphonsathien et al. (2022) [18], Thailand; (4-day group)		
5	Ruggenenti et al. (2022) [15], Italy	Change in glomerular filtration rate (GFR) at 6 months versus baseline.	Other outcomes included anthropometric measurements, blood pressure (BP), changes in albuminuria and albumin fractional clearance, GDR, HbA1c, plasma lipids, hs-CRP, regression from micro- to normo-albuminuria and progression from normo- to micro- and from micro- to macro-albuminuria, remission/regression and new onset or progression of diabetic retinopathy or maculopathy, incidence of major fatal and nonfatal cardiovascular events, health-related quality of life evaluated with the 36-Item Short Form Quality of Life Questionnaire.
6	Ruggenenti et al. (2017) [14], Italy	Change in GFR	Glucose disposal rate (GDR), blood pressure, heart rate, blood glucose, HbA1c, serum lipids (HDL, LDL, triglycerides), plasma renin activity, C-reactive protein (CRP), and safety variables such as vital signs, lab tests, and adverse events.
7	Mollentze et al. (2019) [27], South Africa	Remission of diabetes defined as FPG < 5.6 mmol/L and an HbA1c value of ≤ 6.5% at the end of the study without taking any hypoglycemic agents including insulin.	Secondary: Changes in fasting plasma glucose, total insulin dose, HbA1c, body weight, waist circumference, neck circumference, blood pressure, resting pulse rate, 6-minute walking distance, body fat percentage, serum cholesterol levels, highly sensitive CRP, interleukin 6, leptin levels, and 10-year risk of coronary heart disease.
8	Brown et al. (2020) [16], United Kingdom	Weight loss at 12 months.	Secondary outcomes included insulin usage, HbA1c, fasting plasma glucose, fasting plasma C-peptide, hormonal responses during the mixed meal tolerance tests (MMTT), serum lipids, blood pressure, body composition, and quality of life.
9	Gulsin et al. (2020) [17], United Kingdom	Change in left ventricular (LV) peak early diastolic strain rate (PEDSR) as measured by cardiac magnetic resonance (CMR).	Echocardiographic measures of diastolic function (E/A, E/e'); CMR measures of cardiac structure and function Myocardial perfusion reserve; aortic stiffness; peak oxygen uptake (VO2).
10	Hu et al. (2019) [26], China	Change in Angiopoietin-like protein 8 (ANGPTL8) concentration at 6 months.	Changes in weight, BMI, waist, body composition, BP, HbA1C, FPG, fasting insulin, lipid profile, physical activity, drug use, liver fat content, liver and kidney functions, high sensitivity (hs)-CRP.
11	Li et al. (2024) [20], China	Change in glycemic control (HbA1c).	Changes in other glycemic metrics, body weight, body composition, liver fat content, serum lipids, and blood pressure.

**Table 5:** Inclusion and exclusion criteria of the selected studies. T2DM, type 2 diabetes mellitus; HbA1c, glycated hemoglobin; BMI, Body Mass Index; VLCD, very low-calorie diet; ALT, alanine aminotransferase; ULN, upper limit of normal; NSAID, non-steroidal anti-inflammatory drug; NYHA, New York Heart Association; CAD, coronary artery disease; BP, blood pressure; LDL, low-density lipoprotein; MI, myocardial infarction.

S. No.	Study	Inclusion criteria	Exclusion criteria
1	Lean et al. (2018) [25], United Kingdom	Aged 20–65 years; Diagnosed with T2DM within the previous 6 years; BMI between 27–45 kg/m²; Not receiving insulin.	Current insulin use; HbA1c concentration of ≥12% (≥108 mmol/mol); Recent significant weight loss (>5 kg in the past 6 months); Severe or unstable heart failure, known cancer, or recent myocardial infarction; Learning difficulties, eating disorders, or pregnancy considerations; Estimated glomerular filtration rate (eGFR) <30 mL/min/1.732 m²; Participation in another clinical trial; Substance abuse; Current anti-obesity drug treatment; Hospital admission for depression or use of antipsychotic drugs.
2	Yang et al. (2023) [19], China	Age: Participants aged between 18 and 75 years; Type 2 Diabetes Diagnosis: Participants diagnosed with T2DM based on the 1999 World Health Organization recommendations; BMI: Participants with a BMI between 18 and 35 kg/m²; Medication: Participants who were taking T2D medications such as sulfonylureas, meglitinides, metformin, dipeptidyl peptidase-4 inhibitors (DPP4i), glucagon-like peptide-1 agonist (GLP1-RA), thiazolidinedione, and insulin; Cognitive Ability: Participants who could understand and carefully follow study directions.	Previous use of insulin, fasting C-peptide <1 ng/mL; thiazolidinedione or GLP-1 receptor agonist use in the past 3 months; serum creatinine >1.5 mg/dL, ALT >2.5x ULN​.
3	Williams et al. (1998) [13], United States; (1-day group)	Age: Participants aged between 30 and 70 years; Type 2 Diabetes: Participants with type 2 diabetes who were more than 20% over their ideal body weight based on Metropolitan Life Insurance norms; Medication: Participants not currently receiving insulin therapy; Fasting Plasma Glucose (FPG): Participants with FPG levels of less than 16.7 mmol/L after discontinuing oral diabetes medications.	History of liver disease, renal disease, or heart disease that would contraindicate the use of a VLCD; fasting plasma glucose (FPG) levels >16.7 mmol/L after discontinuation of diabetes medications.
	Williams et al. (1998) [13], United States; (5-day group)		
4	Umphonsathien et al. (2022) [18], Thailand; (2-day group)	Age: Participants aged between 30 and 60 years; Diagnosis: Participants diagnosed with type 2 diabetes within the previous 10 years; BMI: Participants with a BMI ≥23 kg/m²; HbA1C: Participants with HbA1C levels between 6.5 and 10%.	Fasting C-peptide level <1 ng/mL; previous use of insulin; previous treatment with thiazolidinedione or glucagon-like peptide-1 (GLP-1) receptor agonist in the past 3 months; serum creatinine more than 1.5 mg/dL; serum alanine aminotransferase (ALT) more than 2.5 times the upper limit of the reference range​.
	Umphonsathien et al. (2022) [18], Thailand; (4-day group)		
5	Ruggenenti et al. (2022) [15], Italy	Age: Participants aged over 40 years; Diagnosis: Participants with T2DM; BMI: Participants with a BMI of 27 kg/m² or greater; Serum Creatinine: Participants with serum creatinine levels less than 1.2 mg/dL; Albuminuria: Participants with urinary albumin excretion (UAE) of 300 mg/24 h or less; Stable Diet and Medication: Participants with no systematic changes in calorie, protein, sodium intake, or treatment with blood pressure, glucose, or lipid-lowering agents over the last 3-6 months.	Concomitant non-diabetic kidney disease; urinary tract infection; ischemic kidney disease; treatment with steroids or NSAIDs; heart failure; uncontrolled diabetes; hypo/hypernatremia; prior bariatric surgery; pregnancy, depression; alcohol or drug abuse​.
6	Ruggenenti et al. (2017) [14], Italy	T2DM Diagnosis: Participants with a diagnosis of type 2 diabetes; Abdominal Obesity: Defined as a waist circumference greater than 94 cm in men and greater than 80 cm in women; Age: Participants aged over 18 years; Serum Creatinine: Levels less than 1.2 mg/dL; Normoalbuminuria: Defined as urinary albumin excretion (UAE) of less than 20 mg/min in overnight urine collections; Stable Body Weight: Participants with stable body weight and calorie intake; Stable Diet: Participants following a standardized diet with a stable intake of micro- and macronutrients and salt, according to guidelines, with no systematic changes in blood pressure, glucose, and lipid-lowering medications during the previous 6 months.	
7	Mollentze et al. (2019) [27], South Africa	Age: Participants aged 35–65 years; Diagnosis: Participants with T2DM diagnosed at least 4 years previously; BMI: Participants with a BMI of ≥35 kg/m²; Weight: Participants with a body weight of less than 185 kg.; Insulin Therapy: Participants who had been on insulin treatment for at least 12 months; HbA1c: Participants with HbA1c levels of ≥6.5%.	Secondary diabetes; advanced renal disease; HIV; malignancies; heart failure (NYHA class >2); unstable angina; untreated major depressive disorder; prior bariatric surgery.
8	Brown et al. (2020) [16], United Kingdom	Age: Participants aged 18–70 years; Diagnosis: Participants with T2DM; Treatment: Participants who were treated with insulin therapy; BMI: Participants with a BMI of ≥30 kg/m².	Participants on insulin therapy for more than 10 years; fasting circulating C-peptide of less than 600 pmol/L; type 1 diabetes; significant diabetes-related microvascular complications; estimated glomerular filtration rate (eGFR) less than 30 mL/min/1.73 m²; clinically diagnosed binge eating disorder​.
9	Gulsin et al. (2020) [17], United Kingdom	Age: Participants aged 18–65 years; Diagnosis: Participants with established type 2 diabetes (T2D) for at least 3 months but diagnosed before the age of 60 years; BMI: Participants with a BMI greater than 30 kg/m² (or greater than 27 kg/m² if of South Asian or Black ethnicity).	T2D duration >12 years; insulin treatment; cardiovascular diseases (e.g., CAD, stroke, peripheral artery disease, heart failure); weight loss >5 kg in 6 months; inability to exercise; participants currently being treated with more than three glucose-lowering medications.
10	Hu et al. (2019) [26], China	Type 2 Diabetes Diagnosis: Diagnosed within ≤8 months; Hemoglobin A1C (HbA1C): Ranging from 6.5 to 9.0%; Age: Participants aged 18 to 70 years; BMI: Between 24 and 40 kg/m²; Body Weight: Participants with a body weight ≤180 kg.	BP >160/100 mmHg; LDL-C >4.0 mmol/L; cardiac diseases (recent MI or unstable angina); diabetic retinopathy; nephropathy; insulin-dependence; pregnancy; mental disorders​​.
11	Li et al. (2024) [20], China	Age: Participants aged 40–70 years; Diagnosis: Participants with a diagnosis of type 2 diabetes within the prior 2 years; BMI: Participants with a BMI ranging from 25.0 to 39.9 kg/m²; HbA1c Levels: Participants with HbA1c levels ranging from 7.0 to 8.9%.	Use of Insulin: Participants who were currently using insulin or had used it within the past 6 months were excluded from the study; Type 1 diabetes; Cardiovascular event in the previous 6 months; Uncontrolled hypertension; Currently completing >75 min of high-intensity exercise or >150 min of moderate-intensity exercise per week; High alcohol intake; Active foot ulcer; Impaired liver or renal function; history of food allergies or bariatric surgery; pregnancy, breastfeeding, or planning pregnancy; conditions making the individual ineligible for trial.

All studies were publicly funded. However, two [16,18] received formula diets and glucometers from corporate sponsors, but the authors stated there was no corporate influence.

All selected studies demonstrate a low risk of bias across all five domains of the RoB-2 tool [24]. Detailed results of the risk-of-bias assessment are available upon request from the author.

Weight Change Results

**Figure 2:**
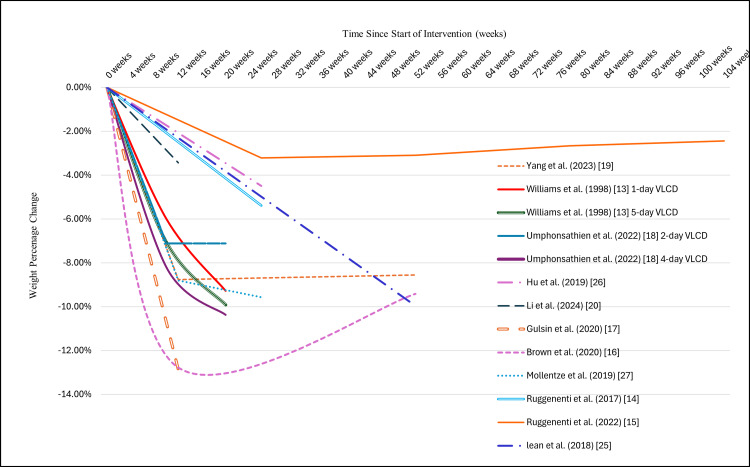
Weight percentage change trend across various calorie restriction interventions. VLCD, very low-calorie diet.

CR led to weight loss exceeding 5% regardless of the CR intervention type and duration. Refer to Figure 2 for weight percentage change trends and Table 6 for weight and BMI changes. The following subsections present the weight change outcomes across various interventions.

**Table 6:** Baseline and change in weight and BMI in the selected studies. WT, weight; INT, intervention group; BMI, Body Mass Index; NR, not reported; SD, standard deviation; SEM, standard error of the mean; 95% CI, 95% confidence interval. * Calculated value; † Mean (SEM); ‡ At 6 months; § At 24 months; || Median BMI change with 95% confidence interval; ¶ Mean (95% CI).

S. No.	Study	Baseline WT mean, kg (SD)	WT change at the end of INT, kg (SD)	WT change at follow-up, kg (SD)	Percentage of WT change		Baseline BMI INT	BMI change INT (Follow-up BMI - Baseline BMI)	
					At the end of INT	At follow-up	Mean, kg/m² (SD)	Mean, kg/m² (SD)	Mean, kg/m² (95% CI)
1	Lean et al. (2018) [25], United Kingdom	100.40 (16.50)	NR	-10 (8.0)	NR	-9.96%^*^	35.00 (4.50)	−3.50 (2.80)	
2	Yang et al. (2023) [19], China	67.60 (6.58)	-5.93 (2.47)	-5.80^* ^(NR)	-8.77%^*^	-8.55%^*^	24.23 (2.58)	−2.41 (1.00)	
3	Williams et al. (1998) [13], United States; (1-day group)	103.50 (16.80)	-9.60 (5.70)		-9.28%^*^		35.40 (5.40)	NR	
	Williams et al. (1998) [13], United States; (5-day group)	104.80 (13.70)	-10.40 (5.40)		-9.92%^*^		37.30 (4.80)		
4	Umphonsathien et al. (2022) [18], Thailand; (2-day group)	77.20 (5.50)^†^	-5.50 (1.30)^†^		-7.12%^*^		29.90 (1.60)^†^	−2.10 (0.50)^†^	
	Umphonsathien et al. (2022) [18], Thailand; (4-day group)	82.90 (5.50)^†^	-8.60 (1.30)^†^		-10.37%^*^		31.00 (1.60)^†^	−3.60 (0.50)^†^	
5	Ruggenenti et al. (2022) [15], Italy	90.20 (11.30)	-2.20^*^ (NR)		-2.44%^*^		32.30 (3.70)		−1.30^*^ (NR)^‡^ −1.00^*^ (NR)^§^
6	Ruggenenti et al. (2017) [14], Italy	87.20 (13.70)	-4.70 (5.5)		-5.39%^*^		30.00 (3.90)	−1.60 (1.90)	
7	Mollentze et al. (2019) [27], South Africa	131.70 (20.51)	-12.60		-9.57%		41.30 (4.41)	−4.00^* ^(NR)	
8	Brown et al. (2020) [16], United Kingdom	104.00 (20.20)	-13.30 (6.8)	-9.8 (4.9)	-12.78%^*^	-9.42%^*^	36.60 (5.10)		−4.60^*^ (NR)
9	Gulsin et al. (2020) [17], United Kingdom	106.70 (16.20)	-13.70^*^		-12.84%^*^		35.20 (33.50 - 40.30)^||^		−4.75 (−5.17 to −4.00)^||^
10	Hu et al. (2019) [26], China	82.40 (NR)	-3.70^*^		-4.49%^*^		29.20 (NR)	–1.44 (NR)	
11	Li et al. (2024) [20], China	74.33 (10.70)	-2.56 (–3.40–1.72)¶		-3.48%^*^		27.59 (2.48)		−0.95 (−1.26 to −0.65)¶

Total diet replacement: Four RCTs investigated the effectiveness of total diet replacement (TDR) interventions for weight loss in T2DM [16,17,25,27]. They had a daily calorie intake of 810 to 878 kcal/day (the 878 kcal value was converted from the corresponding kilojoule figure reported in the study by Mollentze et al. [27]) and variable intervention durations, 12 weeks in two studies [16,17], three to five months (depending on participant preference) in one [25], and six months in another [27]. The reported average weight loss across studies ranged from 10.0 kg (10%) [25] to 13.7 kg (12.8%) [17]. The other studies achieved 12.6 kg (9.6%) [27], and 13.3 kg (12.8%) [16] weight loss. Refer to Table 6 for weight and BMI changes and Figure 2 for weight percentage change trends.

Intermittent calorie restriction: Intermittent VLCD (400-600 kcal/day), when administered for five consecutive days during weeks 2, 7, 12, and 17 (5-day group) [13], led to 9.9% weight loss or 10.4 kg ± 5.4 kg (standard deviation {SD}) [13]. When VLCD was administered for one day per week following five consecutive days of VLCD in the second week (1-day group) [13], it led to 9.3% weight loss or 9.6 kg ± 5.7 kg (SD) [13]. Notably, 93% of the 5-day group participants lost >5 kg during the intervention [13]. Similarly, intermittent VLCD (600 kcal/day) resulted in a 10.4% weight loss or 8.6 kg ± 1.3 kg (SEM) when administered for four non-consecutive days every week for 20 weeks (4-day group) [18] and a 7.1% weight loss or 5.5 kg ± 1.3 kg (SEM) when administered for two non-consecutive days every week for 20 weeks (2-day group) [18]. Likewise, two days per week of intermittent VLCD (790 kcal/day) for 12 weeks resulted in 3.5% weight loss or 2.6 kg (95% confidence interval {95% CI:-3.40-1.72}) [20].

Intermittent LCD of 840 kcal/day, administered for six cycles of five days of LCD followed by ten days of ad libitum diet over 90 days, led to 8.7% weight loss or 5.9 kg ± 2.5 kg (SD), which was maintained at the 12-month follow-up [19].

Continuous calorie restriction: Continuous CR, targeting a 5-10% weight loss through a reduction in the daily calorie intake by 500 kcal, achieved a 4.5% weight reduction or 3.7 kg after six months of intervention [26]. Similarly, a continuous CR intervention aimed to reduce the daily calorie intake by 25% in two studies with different intervention durations [14,15]. They reported a 5.4% weight loss, or 4.7 kg in one study [14], and an initial weight loss of 3.2%, or 2.9 kg in the other study [15] after six months of intervention. However, gradual weight regain was observed from the six-month mark [15]. The mean weight changes from the baseline were 2.8 kg (3.1%), 2.4 kg (2.6%), and 2.2 kg (2.44%) at one year, 18 months, and two years, respectively [15].

Changes in Cardiometabolic Parameters

**Figure 3:**
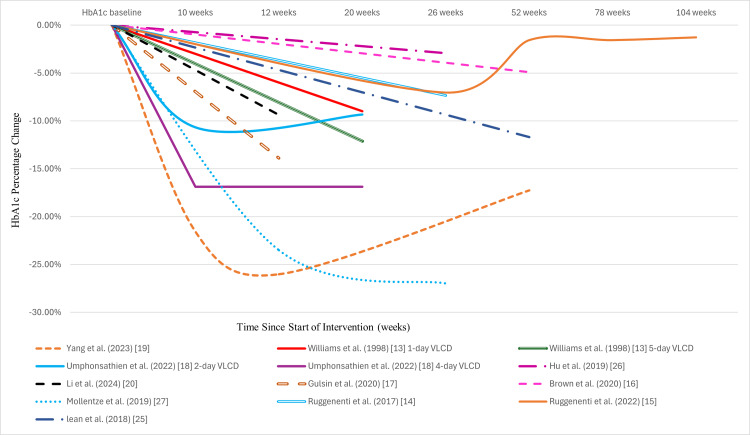
Percentage change in HbA1c levels over time across various calorie restriction interventions. VLCD: very low-calorie diet.

HbA1c dropped with CR to ≤6.5% (48 mmol/mol) in several studies [14,17,19,27], including the 4-day group from the study by Umphonsathien et al. [18]. Moreover, diabetes remission was achieved in a few studies, with a reported remission rate ranging from 19.42% [20] to 83% [17]. Refer to Table 7 for HbA1c changes observed during the intervention and follow-up and Figure 3 for HbA1c percentage change trends.

**Table 7:** HbA1c level changes over time. VLCD, very low-calorie diet; NR, not reported; SEM, standard error of the mean; HbA1c, glycated hemoglobin; SD, standard deviation. * Calculated value; † Mean (SEM).

S. No.	Study	Mean HbA1c level, % (SD)							
		Baseline	10 weeks	12 weeks	20 weeks	6 months	12 months	18 months	24 months
1	Yang et al. (2023) [19], China	7.65 (1.41)		5.66 (0.58)			6.33 (0.87)		
2	Williams et al. (1998) [13], United States; 1-day VLCD	7.90 (1.50)			7.19^*^ (NR)				
3	Williams et al. (1998) [13], United States; 5-day VLCD	8.00 (1.70)			7.03^*^ (NR)				
4	Umphonsathien et al. (2022) [18], Thailand; 2-day VLCD	7.50 (0.30)^†^	6.70 (0.2)^†^		6.80 (0.20)^†^				
5	Umphonsathien et al. (2022) [18], Thailand; 4-day VLCD	7.70 (0.30)^†^	6.40 (0.2)^†^		6.40 (0.30)^†^				
6	Hu et al. (2019) [26], China	7.85 (NR)				7.62 (NR)			
7	Li et al. (2024) [20], China	7.67 (0.82)		6.95^*^ (NR)					
8	Gulsin et al. (2020) [17], United Kingdom	7.20 (1.10)		6.20 (0.70)					
9	Brown et al. (2020) [16], United Kingdom	8.75 (1.74)					8.32^*^ (NR)		
10	Mollentze et al. (2019) [27], South Africa	8.90 (1.74)		6.80 (0.65)		6.50 (0.64)			
11	Ruggenenti et al. (2017) [14], Italy	6.80 (1.00)				6.30 (0.70)			
12	Ruggenenti et al. (2022) [15], Italy	7.10 (3.10)				6.60 (3.00)	6.90 (3.30)	6.90 (3.30)	7.10 (3.30)
13	Lean et al. (2018) [25], United Kingdom	7.70 (1.20)					6.80 (1.20)		

Lipid parameters were reported by all studies except two [17,19]. The triglyceride levels decreased in all the studies; however, the reduction was statistically significant in only four [15,18,25,26]. High-density lipoprotein (HDL) levels increased in most of the studies [15,16,20,25-27], including the 1-day group of the study by William et al. [13] and the 4-day group of the study by Umphonsathien et al. [18]. However, the increase was statistically significant in only four [14,20,25,27].

The changes in low-density lipoprotein (LDL) levels were mixed and statistically insignificant. While its levels decreased in some studies [13-15,26], it increased in others [16,18,20,27]. Similarly, the changes in total cholesterol levels were statistically insignificant. Nevertheless, its levels dropped in all studies except two [18,25].

A statistically significant reduction in systolic and diastolic BP was observed following a 25% reduction in daily calorie intake for six months or two years [14,15]. Additionally, some studies showed a significant reduction in either systolic [17,18], or diastolic BP [20]. On the other hand, several studies [16,19,25-27] found no significant change in the systolic or diastolic BP in response to CR. Refer to Table 8 for details of the observed changes in systolic BP.

**Table 8:** Systolic blood pressure change with intervention. SD, standard deviation; NR, not reported; (95% CI), 95% confidence interval. NS, non significant, P value is not significant; BP, blood pressure. * P value intervention vs control; † p < 0.025 considered significant; ‡ mmHg (95% CI); § P value is a comparison of the difference between the CMNT (Chinese medical nutrition therapy) group and control group from baseline to 3 months; || P value is a comparison between 3-month and 12-month follow-up for both CMNT and control groups.

S. No.	Study	Time of measurement	Systolic BP, mmHg (SD)	P value
1	Williams et al. (1998) [13], United States; (1-day group)	NR		
2	Williams et al. (1998) [13], United States; (5-day group)	NR		
3	Brown et al. (2020) [16], United Kingdom	Baseline	131.5 (16.1)	
		Change at 12 months	−7.7 (15.0)	0.76
4	Ruggenenti et al. (2017) [14], Italy	Baseline	127.8 (9.7)	
		6 months	121.1 (9.9)	0.0322^*^
5	Umphonsathien et al. (2022) [18], Thailand; (2-day group)	Baseline	122.9 (5.1)	
		10 weeks	128.3 (4.6)	0.275
		20 weeks	121.7 4.6	0.794
6	Umphonsathien et al. (2022) [18], Thailand; (4-day group)	Baseline	140.9 (5.1)	
		10 weeks	127.4 (4.6)	0.008
		20 weeks	131.1 (3.7)	0.042
7	Hu et al. (2019) [26], China	Baseline	135 (NR)	
		6 Months	134 (NR)	
		P value	0.3109^†^	
8	Gulsin et al. (2020) [17], United Kingdom	Baseline	145.9 (15.9)	NR
		week 12	132.9 (18.0)	NR
9	Li et al. (2024) [20], China	12 weeks−Baseline	−1.04 (−4.89 to 2.82)^‡^	0.093
10	Lean et al., (2018) [25], United Kingdom	Baseline	134.3 (17.6)	
		12 months	133.0 (16.3)	
		P value	0.771	
11	Ruggenenti et al. (2022) [15], Italy	Baseline Mean (SD)	132.9 (11.6)	NS
		6 months	127.3 (11.0)	<0.05
		12 months	131.7 (12.1)	NS
		18 months	130.8 (12.2)	NS
		24 months	132.0 (12.4)	NS
12	Yang et al. (2023) [19], China	Baseline	129.41 (6.94)	
		3 months	128.66 (6.87)	0.519^§^
		12 Months	129.10 (5.24)	0.77^||^
13	Mollentze et al. (2019) [27], South Africa	Baseline	133.6 (12.5)	NS
		3 Months	132.22 (18.5)	NS
		6 Months	131.3 (18.1)	NS

Discussion

Weight Loss Outcome

CR, implemented through various interventions and over different durations, resulted in significant weight loss. Of the 11 studies reviewed, eight [13,14,16-19,25,27] achieved a weight reduction of ≥5%. Moreover, three studies achieved weight losses exceeding 10%: the 4-day group from the study by Umphonsathien et al. [18], and the studies by Brown et al. [16] and Gulsin et al. [17]. These outcomes align with the findings of a recent systematic review, which reported similar weight losses with CR interventions [7].

The effectiveness of CR is closely linked to both its intensity and duration. The most rapid and substantial weight loss was observed with TDR interventions in the studies by Brown et al. and Gulsin et al., both reporting weight reductions exceeding 12% [16,17]. A similar outcome was anticipated in the study by Lean et al. and Mollentze et al., as they also implemented TDR interventions [25,27]. However, the study by Lean et al. did not report weight changes at the end of the intervention, when the most significant drop in weight likely would have occurred [25]. In the study by Mollentze et al. [27] participants had higher baseline BMI, making the percentage of weight change appear smaller, despite achieving similar absolute weight loss to that seen in the studies by Brown et al. [16] and Gulsin et al. [17]. Additionally, the higher average daily calorie intake in the study by Mollentze et al. likely limited weight reduction [27].

Intermittent CR, with VLCD of 400-790 kcal/day [13,18,20], or an LCD of 840 kcal/day [19], resulted in weight loss ranging from 3.5% to 10.4%. Notably, the weight loss reported in the study by Li et al. [20] deviated from that reported by other studies [13,18,19]. Variations in CR intensity, shorter durations, or fewer calorie-restricted days likely explain this heterogeneity. Since population characteristics, baseline BMI, and medication use were consistent across studies, these factors are unlikely to be confounding variables.

Although VLCD is shown to achieve substantial weight loss, it is important to note that VLCD can lead to significant muscle mass loss. This was reported by a recent systematic review [28].

Continuous CR, achieved by a 25% reduction in daily calorie intake [14,15], or a daily reduction of 500 kcal to target a 5-10% weight loss [26], led to relatively modest weight loss (3.2-5.4%) [14,15,26]. This approach proved to be the least effective among the interventions reviewed. A possible explanation is that participants did not consistently achieve the intended 25% calorie reduction. For instance, one study [14] reported a 15% reduction, while the other [15] observed a less than 20% reduction. Notably, the study [26] that targeted a 500 kcal deficit did not explicitly confirm if it was achieved. Another possible reason is reduced motivation to adhere to CR with longer interventions.

Several alternatives to CR exist for weight loss. One is bariatric surgery, which can lead to substantial weight loss, with reductions of up to 80% of excess body weight within one year [29]. However, an RCT [30] reported that a VLCD of approximately 500 kcal/day could yield comparable short-term results to surgery. Medications are another alternative, particularly glucagon-like peptide-1 receptor agonists (GLP1 RAs); a systematic review reported up to 17% weight loss after a year [31]. However, GLP1 RA effectiveness is reduced in individuals with T2DM [31]. Additionally, low-carbohydrate diets were assessed for weight loss in T2DM, but a recent review found them ineffective [32]. In conclusion, CR offers a practical alternative for weight loss in individuals with T2DM. It has benefits such as lower cost, reversibility, and reduced risks compared to surgery, as well as being safe [7] and injection-free compared to GLP1 RAs.

Cardiometabolic Parameters

CR reduces cardiovascular risk by improving cardiometabolic parameters. It is associated with reductions in HbA1c, triglyceride level, systolic and diastolic BP, and an increase in HDL level. Changes that are associated with cardiovascular risk reduction [33-35].

A reduction in HbA1c to ≤6.5% (48 mmol/mol) was observed in studies that reported significant weight loss, specifically those achieving >8% weight reduction and >2.10 kg/m² BMI reductions. Likewise, this group achieved a high diabetes remission rate of 83% [17], 47% [19] and 29% [18]. However, three studies [13,16,25] with comparable weight loss did not observe HbA1c levels falling to ≤6.5% (48 mmol/mol). This heterogeneity may be attributed to several factors. First, the studies by Lean et al. [25] and Brown et al. [16] discontinued antidiabetic medications at the start of the intervention, potentially losing their HbA1c-dropping effects. Second, 39% of participants in the intervention group of the study by Brown et al. discontinued insulin, losing its effect on HbA1c [16]. Lastly, HbA1c levels were measured at the 12-month mark, long after the intervention had ended [16,25]. Potentially not capturing the initial HbA1c reduction, which gradually increases over time, either during the intervention [15,18] or follow-up [19]. Therefore, these factors likely contributed to underestimating the actual HbA1c reductions achieved by the interventions. Nevertheless, the study by Lean et al., despite not reporting an HbA1c reduction below 6.5%, has achieved a 46% diabetes remission [25]. Refer to Table 7 for HbA1c changes and Figure 3 for HbA1c percentage change over time.

The explanation why the third study [13] failed to achieve an HbA1c reduction to ≤6.5% (48 mmol/mol) despite the achieved weight reduction remains unclear. However, it is noteworthy that over 31% of participants in the intervention group achieved an HbA1c level <6.0% (42 mmol/mol) in this study [13].

In contrast, studies reporting weight reductions of ≤8% and BMI reduction of ≤2.10 kg/m² failed to achieve an HbA1c level of ≤6.5% (48 mmol/mol). Additionally, these studies demonstrated lower diabetes remission rates, with one reporting a rate of 19% [20]. Interestingly, one study [14] observed an HbA1c reduction below 6.5% (48 mmol/mol) despite achieving only a modest weight loss of 2.4% and a BMI reduction of 1.60 kg/m². This outcome can be attributed to the study’s low baseline HbA1c levels, which allowed the target to be reached with a small reduction.

The definition of diabetes remission differed across the studies; however, they all shared the standard of achieving HbA1c levels below 6.5% (48 mmol/mol) after discontinuing all antidiabetic medications. While disagreeing on the required duration to sustain remission. Refer to Table 9 for the definition of diabetes remission for each study.

**Table 9:** Definition of diabetes remission in the selected studies. T2DM, type 2 diabetes mellitus; HbA1c, glycated hemoglobin.

S. No.	Study	Definition
1	Gulsin et al. (2020) [17], United Kingdom	Fasting glucose of <7.0 mmol/L or HbA1c <6.5% without taking any hypoglycaemic agent postintervention were considered to have remission of T2DM.
2	Yang et al. (2023) [19], China	Stable HbA1c level of less than 48 mmol/mol (< 6.5%) for at least three months after discontinuing all antidiabetic medications.
3	Lean et al. (2018) [25], United Kingdom	HbA1c of less than 6.5% (<48 mmol/mol) after at least two months off all antidiabetic medications, from baseline to 12 months.
4	Umphonsathien et al. (2022) [18], Thailand	Fasting plasma glucose level <126 mg/dL and HbA1c level <6.5% in the absence of pharmacological therapy for diabetes at the end of the study.
5	Li et al. (2024) [20], China	HbA1c <6.5% without antihyperglycemic medication after the intervention.

These findings suggest that reductions in HbA1c and diabetes remission rates are closely linked to the degree of weight loss, with studies showing more substantial reductions in HbA1c and higher remission rates achieving more significant weight loss. This aligns with existing evidence indicating a positive correlation between weight loss and reduction in HbA1c [5-7,11].

Lipid parameters show favorable changes with CR, with the most consistent finding across studies being reduced triglyceride levels. CR also led to a significant increase in HDL levels in several studies [14,20,25,27]. Both lower triglyceride levels and higher HDL are associated with a reduced risk of cardiovascular disease [33-35].

The study by Lean et al. was the only one to report both a significant reduction in triglyceride and a significant increase in HDL [25]. Notably, this study had the largest intervention group, which may have contributed to the statistical significance of its findings.

The changes in LDL and total cholesterol levels were mixed, with some studies reporting increases while others observed decreases (as presented in the Results section). However, these mixed outcomes, along with the reduction in triglyceride and increase in HDL, align with existing evidence from a recent meta-analysis [7]. Interestingly, this meta-analysis demonstrated that LDL and total cholesterol initially decrease with CR, but this trend reverses as calorie intake falls below 1,500 kcal/day. In contrast, triglyceride levels continue to decline while HDL levels continue to increase with more intensive CR [7].

CR is linked to reductions in systolic, diastolic, or both BP values [14,15,17,18,20]. However, some studies reported BP reductions that were not statistically significant [16,19,25-27]. The reason for the lack of statistically significant differences in BP is not immediately apparent. However, it reflects existing evidence, as some studies have demonstrated significant BP reductions with CR [7,11], while others found no significant difference [36].

Limitations

This review included eleven studies with a low risk of bias. The limitations identified in the studies are listed below:

Missing data and data gap: Some studies did not report specific outcomes relevant to this review, such as BP [13], BMI [13], and lipid parameters [17,19]. Efforts were made to retrieve this data from supplementary materials. The missing data is acknowledged in the text and tables.

There is a lack of long-term studies on CR’s effectiveness for weight loss in T2DM. Only one study [15] employed a two-year intervention. This limited the review’s ability to assess the long-term effectiveness of CR for weight loss.

Population factors: The studies involved participants from diverse geographical and ethnic backgrounds, improving generalizability but introducing heterogeneity. Variation was noted in antidiabetic medication use: some discontinued the antidiabetic medications [25], including insulin [16], while others either excluded insulin users [17,20,25,26] or focused solely on participants using insulin [18,27].

The study by Mollentze et al. [27] included only male participants, limiting its generalizability, as male individuals tend to lose more weight than female individuals with similar interventions [13]. See Table 2 for gender distribution and Table 5 for the inclusion and exclusion criteria of the selected studies.

Intervention variability: The CR intervention varied, including VLCDs [13,18,20], LCD [14,15,19,26], and TDR protocols [16,17,25,27], which were implemented either continuously [14,15,26] or intermittently [13,18-20]. This variability complicates direct comparisons.

Outcome factors: Only three studies [13,16,25] identified weight loss as the primary outcome, potentially affecting the consistency of weight change reporting. A summary of the outcome measures is provided in Table 4.

## Conclusions

In conclusion, CR is an effective intervention for weight loss in individuals with T2DM, with TDR being the most effective method among the CR interventions studied. Moreover, CR reduces cardiovascular risk by improving cardiometabolic factors such as glycaemic control, lipid parameters, and BP. These findings reinforce the potential of CR as a viable and impactful strategy in managing both weight and cardiovascular risk in T2DM patients. Updating clinical guidelines to include CR as a main tool to induce weight loss in patients with T2DM. In addition to training the clinicians on the safe application of CR, in order to broaden the safe use of CR in practice is needed. Finally, long-term studies addressing CR in T2DM are needed to understand its effectiveness better and confirm the long-term sustainability of its weight loss benefits.

Based on the results of the reviewed studies, the author speculates that using cycles of short periods of intense CR, like those used in the TDR studies, followed by a period of ad libitum diet, like the approach used in the intermittent CR studies, might offer a more sustainable method for achieving substantial weight loss. Therefore, the author proposes conducting an RCT to test this hypothesis. The study will use CR of 800-900 kcal/day for four weeks, followed by eight weeks of an ad libitum diet over a period of two years.

## Appendices

**Table 10:** Data extraction template.

Category	Details
Study information	Author(s), year of publication, title of the study, journal name, country
Study design	Study type
	Duration of the study
	Setting (e.g., clinical, community-based)
Participant characteristics	Total number of participants
	Age range or mean age
	Gender distribution
	BMI range or mean BMI at baseline
	Inclusion criteria
	Exclusion criteria
Intervention details	Description of intervention
	Duration of the intervention
	Dietary advice/restrictions
Comparator group (if applicable)	
Outcome measures	Primary outcome(s) assessed
	Secondary outcome(s) assessed
	Method of outcome assessment (e.g., measured, self-reported)
Results	Summary of key findings related to weight loss and other relevant outcomes
	Effect sizes or relative risks
	Adverse events or side effects reported
Quality sssessment	Summary of quality assessment for each included study
Comments/notes	
